# Supplementary figures and images for: Simulation models of dengue transmission in Funchal, Madeira Island: Influence of seasonality
Source: PLoS Negl Trop Dis. 2020 Oct 5;14(10):e0008679. doi: 10.1371/journal.pntd.0008679 (PMC7561266; doi:10.1371/journal.pntd.0008679)

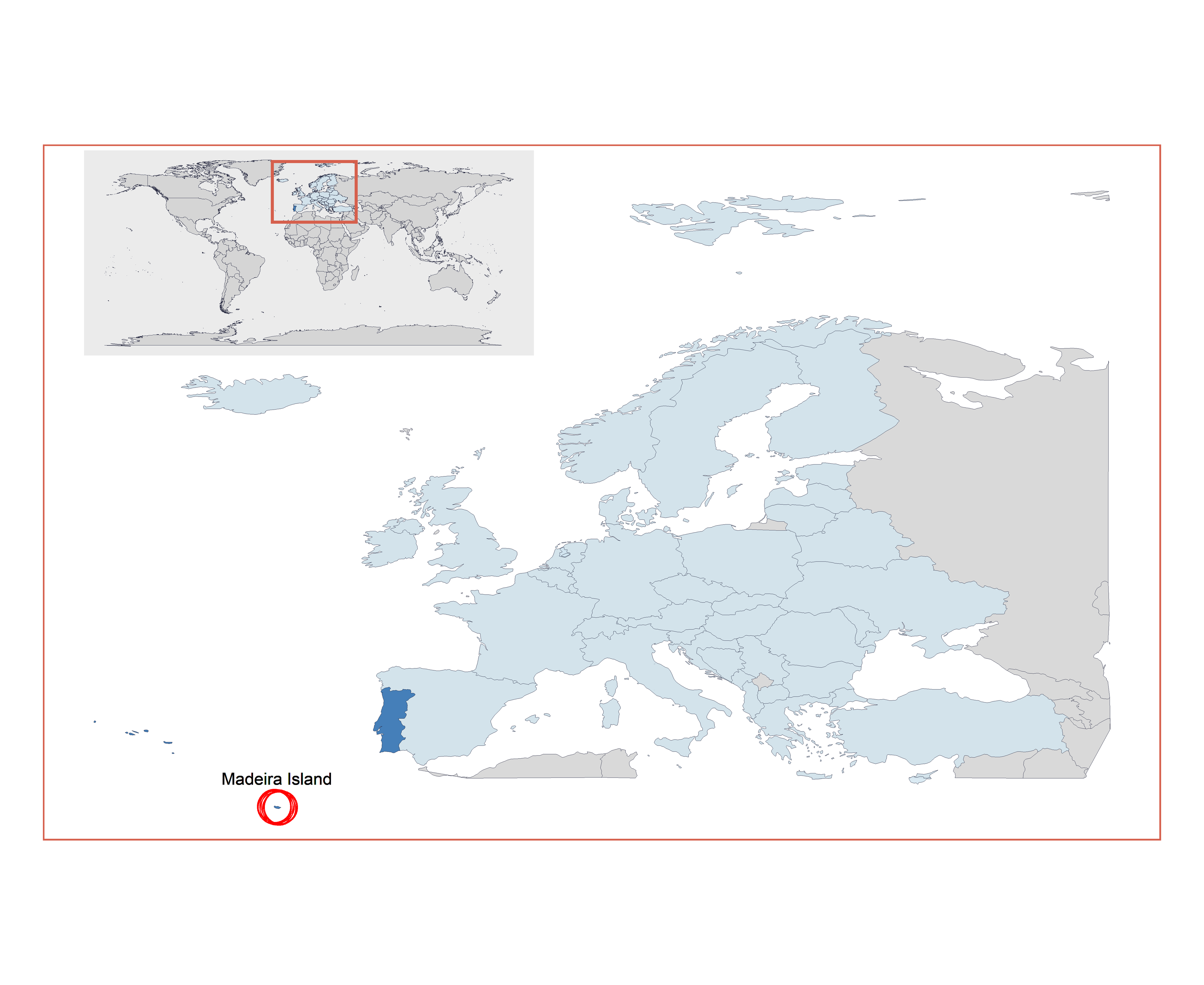

Supplement: S1 Fig — Map of Europe in light blue, Portugal and Azorean islands in dark blue, Madeira island circled in red. (TIF) [file pntd.0008679.s001.tif]

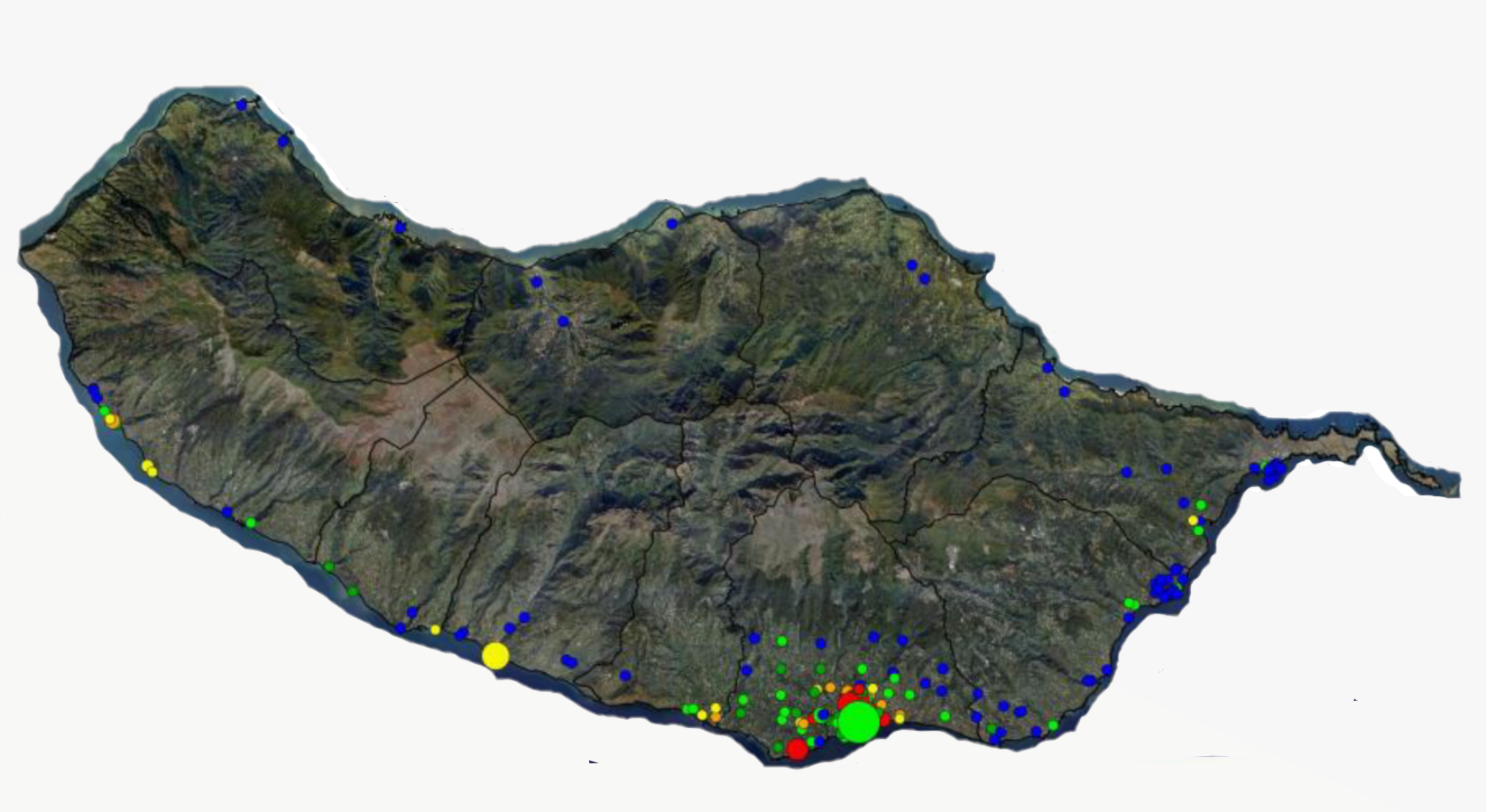

Supplement: S2 Fig — The distribution of ovitrap location across the island, as reported in the Entomological panel bulletins (PEnt_RAMW52/2019, in Portuguese) of the Institute of Health Administration, IP-RAM (IASAÚDE IP-RAM). The colored dots on the map, represents the cumulative number of eggs in each location for the week 10, 2018 to week 52, 2019. Blue = 0%, Dark green = 1%-10%, Light green = 11%–20%, Yellow = 21%–30%, Orange = 31%–40%, Red = 41%–100%. Cumulative number of eggs for all location = 37766. Adapted from Institute of Health Administration, IP-RAM [34]. (TIF) [file pntd.0008679.s002.tif]

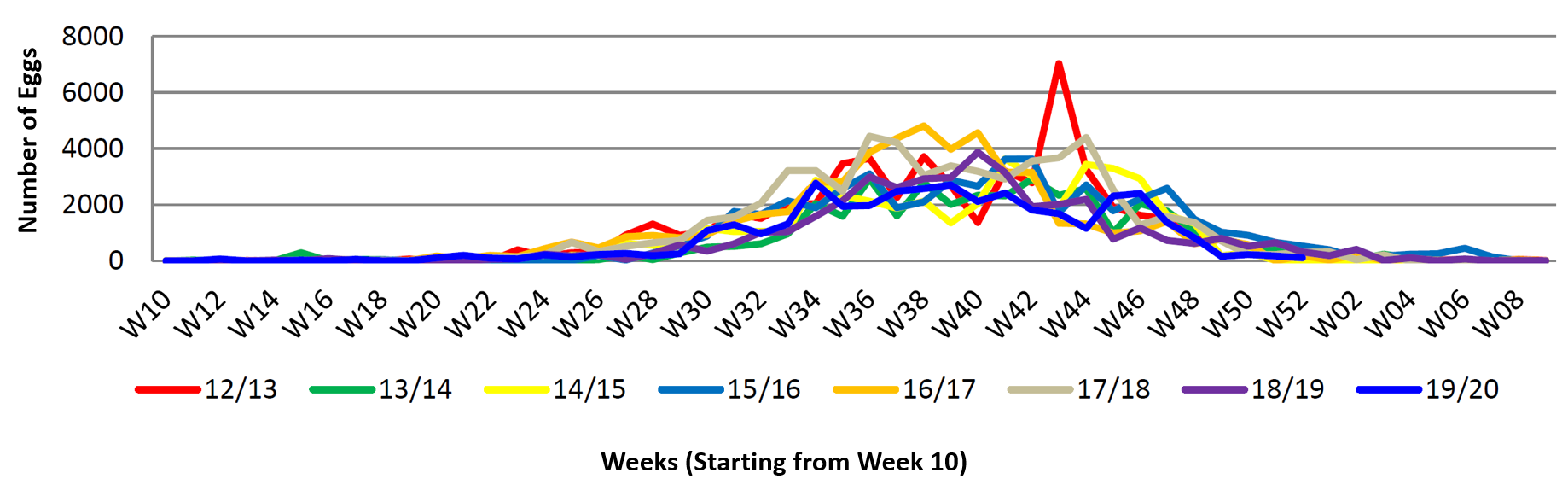

Supplement: S3 Fig — Annual cumulative number of eggs reported across the island from all the ovitraps, as reported in the Entomological panel bulletins (PEnt_RAMW52/2019, in Portuguese) of the Institute of Health Administration, IP-RAM (IASAÚDE IP-RAM). Entomological year starts from week 10. Graphs shows cumulative number of eggs for week 10, 2012 to week 52, 2019, with each entomological year represented in a different line color. Adapted from Institute of Health Administration, IP-RAM [34]. (TIF) [file pntd.0008679.s003.tif]

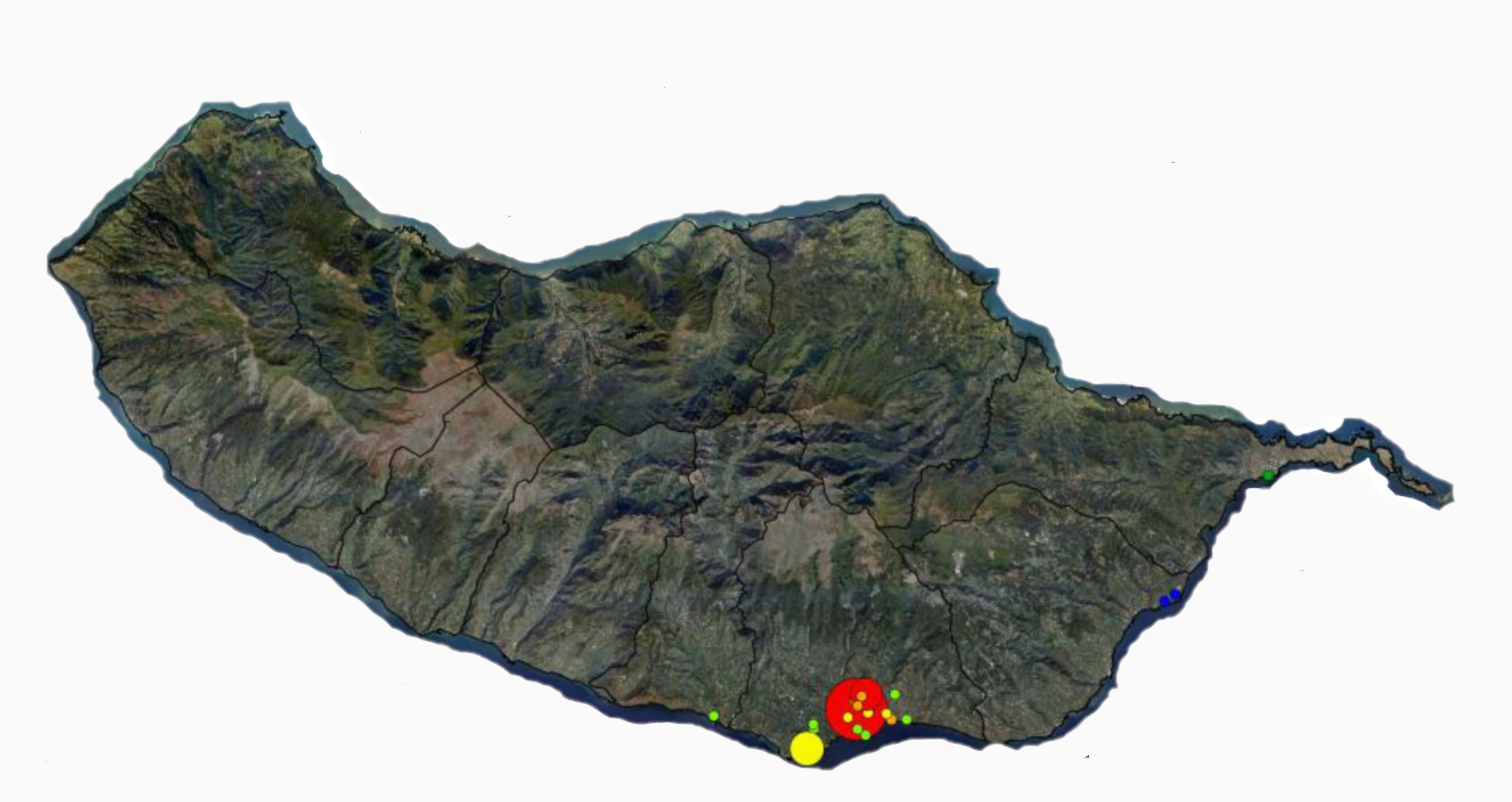

Supplement: S4 Fig — The distribution of BG-traps location across the island, as reported in the Entomological panel bulletins (PEnt_RAMW52/2019, in Portuguese) of the Institute of Health Administration, IP-RAM (IASAÚDE IP-RAM). The colored dots on the map, represents the cumulative number of adult mosquitoes in each location for the week 10, 2018 to week 52, 2019. Blue = 0%, Dark green = 1%-10%, Light green = 11%–30%, Yellow = 31%–50%, Orange = 51%–60%, Red = 61%–100%. Cumulative number of adult mosquitoes for all location = 3464. Adapted from Institute of Health Administration, IP-RAM [34]. (TIF) [file pntd.0008679.s004.tif]

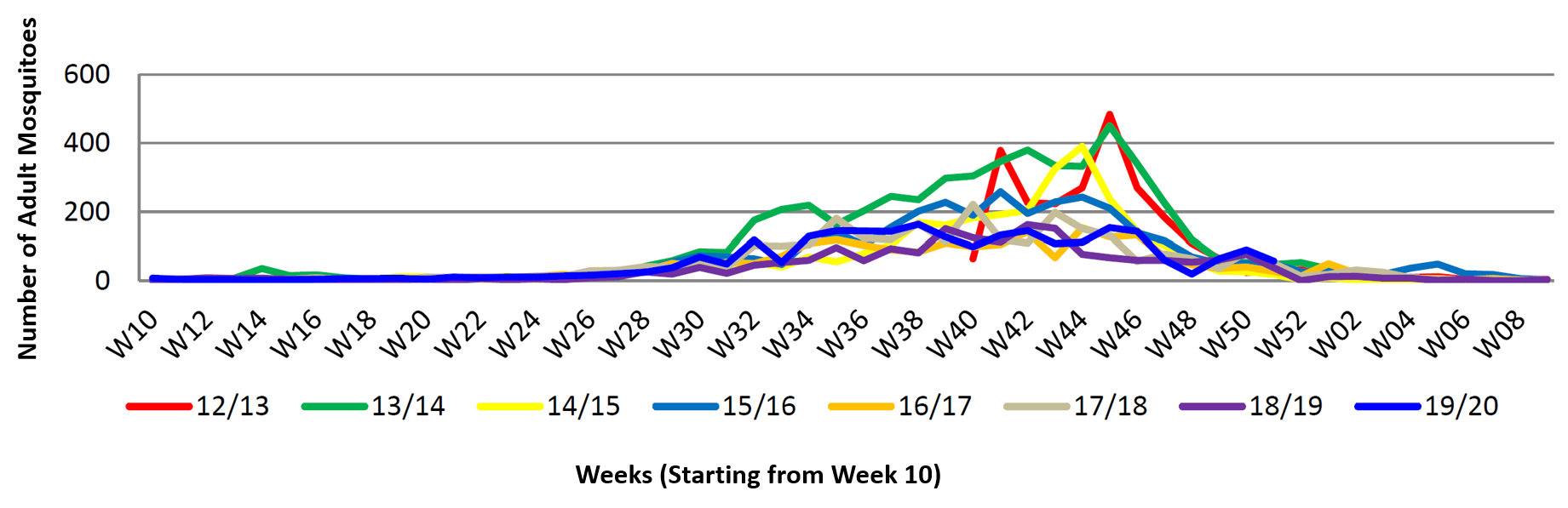

Supplement: S5 Fig — Annual cumulative number of adult mosquitos reported across the island from all the BG-traps, as reported in the Entomological panel bulletins (PEnt_RAMW52/2019, in Portuguese) of the Institute of Health Administration, IP-RAM (IASAÚDE IP-RAM). Entomological year starts from week 10. Graphs shows cumulative number of eggs for week 40, 2012 to week 52, 2019, with each entomological year represented in a different line color. Adapted from Institute of Health Administration, IP-RAM [34]. (TIF) [file pntd.0008679.s005.tif]

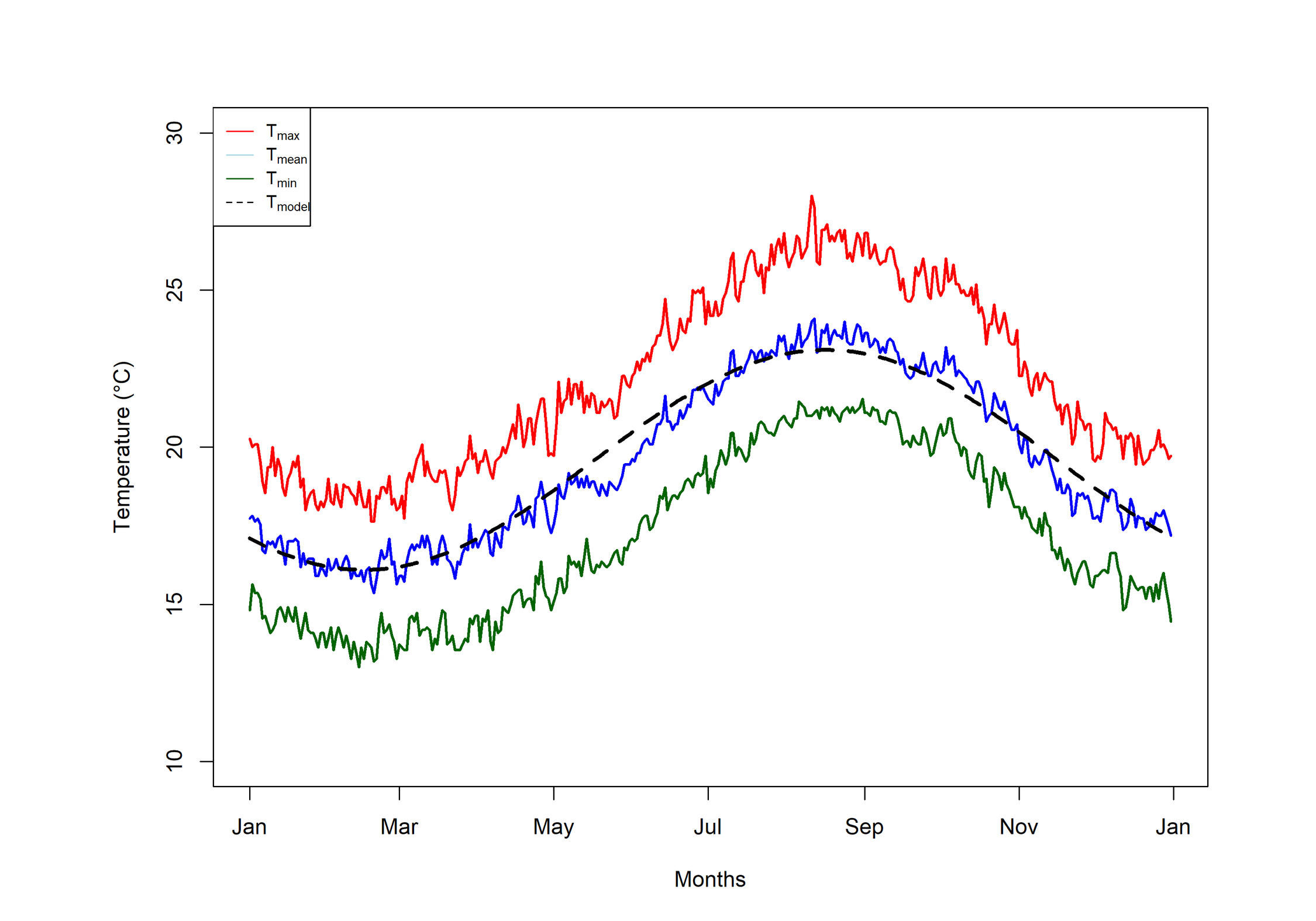

Supplement: S6 Fig — The temperature seasonality pattern as observed from the historical decadal data for Funchal, Madeira. Average of minimum (green line), mean (blue line) and maximum (red line) temperatures per day between 2008 and 2018. Black dashed line is daily mean temperature of our model as calculated from sinusoidal curve (Eq 16 in the main article). The long-term average conditions in Funchal, Madeira Tmax = 22°C, Tmean = 20°C, Tmin = 17°C. Average mean temperatures for the spring months (Mar–May) was 18°C; Summer months (Jun–Aug) was 22°C; Autumn months (Sep–Nov) was 21°C and Winter months (Dec–Feb) was 17°C. (TIF) [file pntd.0008679.s006.tif]
