# Supplementary material for: Simulation models of dengue transmission in Funchal, Madeira Island: Influence of seasonality
Source: PLoS Negl Trop Dis. 2020 Oct 5;14(10):e0008679. doi: 10.1371/journal.pntd.0008679 (PMC7561266; doi:10.1371/journal.pntd.0008679)
Supplement: S1 File — Model starting conditions. (DOCX) [file pntd.0008679.s007.docx]

## **Simulation models of dengue transmission in Funchal, Madeira Island: influence of seasonality**

Donald Salami, César Capinha, Carla Alexandra Sousa, Maria do Rosário Oliveira Martins, Cynthia Lord

## **S1 Appendix.**

## **Model starting conditions**

The model requires an estimate of each state variable (humans and mosquitoes in each class), along with estimates or values chosen from distributions for each parameter. Our parameter values are based on multiple citations from reviewed literature of previous empirical studies or lab trials and expert opinion (Table 2, of the main article). We emphasize previous studies within the context of Funchal, Madeira Island (since our main objective was to parameterize the model for the island) and chose parameter ranges to reflect conditions in Funchal.

The model assumes a homogeneously mixed population, with a total human population set at a constant 30,000 (representative of the population of the most populous civil parish –*Santo António* – in the municipality of Funchal and the island) [1]. Since the human components of the transmission cycle are not seasonal, we set the intrinsic incubation ($1/{\gamma_{h}}$) and the infectious period ($1/{\eta_{h}}$) to constants of 6 and 4 days respectively. For this model, we considered only a single dengue serotype. However, these constants reflect mean values for DENV-2 and DENV-4 as reported in previous literature [2-6]. Based on our mosquito recruitment term, the initial susceptible mosquito population is set as the number of females ($\rho_{b}\left( t \right)$) added to the population at intervals ($iv$), as calculated in equation (12) in the main article. With all infectious classes for both human and mosquitoes set to zero, an infection is triggered by the arrival of one infectious human on a specified day ($t_{crit}$) into the fully susceptible population. The default initial conditions are thus: ($S_{h}; E_{h}; I_{h}; R_{h}; S_{v}; E_{v}; I_{v}$) = ($N_{h}; 0; 0; 0; p_{b}\left( t \right); 0; 0$) where $N_{h}=30,000$.

Simulations were set to start at the coldest day in the annual cycle (i.e. February 15) and ran for 730 days thereafter (allowing for simulation with $t_{crit}$later in the year). We set an arbitrary cut-off value for the exposed and infectious classes (i.e. human and mosquitoes): if (${E_{h}, I}_{h} ,{E_{v} , I}_{v}$, all $<$0.5) the simulation is terminated and restarted with the classes set to zero. This cutoff is necessary otherwise extremely low levels of infection may persist for long periods; in the natural system, there would be a high probability that the virus would go extinct [7].

We performed a preliminary exploration of parameter values to determine their effects on transmission. The choice of the final parameter values was based on permissibility for transmission. The preliminary exploration also informed the initial conditions (described above) and parameter ranges for sensitivity analyses (Table 2). Model simulations were performed using the governing systems of differential equations of MATLAB’s inbuilt routine “ode45” [8]. Simulation outputs were analyzed in the R Programming Language version 3.5.3 [9].

# **References**

1. Santo António Parish Council. History of Santo António parish council (in Portuguese): junta de freguesia de Santo António- an autonomous region of Madeira; 2018 [17 Jul 2019]. Available from: <http://www.jf-santoantonio.pt/historia>.

2. Lourenco J, Recker M. Dengue serotype immune-interactions and their consequences for vaccine impact predictions. Epidemics. 2016;16:40-8. doi: <https://doi.org/10.1016/j.epidem.2016.05.003>.

3. Nishiura H, Halstead SB. Natural history of dengue virus (DENV)-1 and DENV-4 infections: reanalysis of classic studies. ‎J Infect Dis. 2007;195(7):1007–13. doi: <https://doi.org/10.1086/511825>.

4. Chan M, Johansson MA. The incubation periods of dengue viruses. PloS One. 2012;7(11):e50972. doi: <https://doi.org/10.1371/journal.pone.0050972>.

5. Gubler DJ, Suharyono W, Tan R, Abidin M, Sie A. Viraemia in patients with naturally acquired dengue infection. Bull World Health Organ. 1981;59(4):623-30. <https://www.ncbi.nlm.nih.gov/pubmed/6976230>

6. Vaughn DW, Green S, Kalayanarooj S, Innis BL, Nimmannitya S, Suntayakorn S, et al. Dengue viremia titer, antibody response pattern, and virus serotype correlate with disease severity. ‎J Infect Dis. 2000;181(1):2-9. doi: <https://doi.org/10.1086/315215>

7. Lord CC, Day JF. Simulation studies of st. Louis encephalitis and west nile viruses: the impact of bird mortality. Vector Borne Zoonotic Dis. 2001;1(4):317-29. doi: <https://doi.org/10.1089/15303660160025930>.

8. MathWorks. MATLAB and Statistics Toolbox Release 2016a. Natick, Massachusetts: The MathWorks Inc.; 2016.

9. R-Core-Team. The R project for statistical computing: CRAN; 2019. Available from: <https://www.r-project.org/>.
