# Supplementary material for: Simulation models of dengue transmission in Funchal, Madeira Island: Influence of seasonality
Source: PLoS Negl Trop Dis. 2020 Oct 5;14(10):e0008679. doi: 10.1371/journal.pntd.0008679 (PMC7561266; doi:10.1371/journal.pntd.0008679)
Supplement: S2 File — Model sensitivity analysis and multi-model inference analysis. (DOCX) [file pntd.0008679.s008.docx]

## **Simulation models of dengue transmission in Funchal, Madeira Island: influence of seasonality**

Donald Salami, César Capinha, Carla Alexandra Sousa, Maria do Rosário Oliveira Martins, Cynthia Lord

## **S2 Appendix.**

## **Model sensitivity analysis**

To characterize the model parameters exerting the most influence on our quantities of interest, we performed a variance-based global sensitivity analysis, using a combination of Latin hypercube sampling (LHS) and a multi-model inference on regression-based models. LHS is a stratified Monte Carlo sampling technique, where specified parameter distributions are divided into (*M)* equiprobable intervals, and then sampled, here (*M*) is the sample size. The entire range of each parameter is explored, by sampling each interval for each parameter only once without replacement. Parameters values are then randomly resorted into sets to use for simulation. The LHS method assumes that the sampling is performed independently for each parameter, thereby allowing for an unbiased estimate [1, 2]. Parameter ranges for sampling were derived from existing literature, expert opinion, and field-based data (Table 2 of the main article), we assumed a uniform distribution for all parameter values.

Multi-model inference on a generalized least square (GLS) regression was used to estimate the relative importance of the input parameters. Multi-model inference uses the information-theoretic approach to offer a more objective way to assess the relative importance of input variables by inferring all possible models from a defined candidate set [3, 4]. A vector of the input parameters ($x_{p})$ were fitted into all possible unique models and then ranked from best to worst, based on Akaike information criterion (AIC) values. An estimate of the relative importance of a single parameter ($p$) was then calculated by summing the Akaike weights ($w)$ across all fitted models where parameter ($p$) occurs. Akaike weights are normalized, such that the sum over all models considered is 1. The relative importance of parameter ($p$) was quantified by the sum $(w$) for the parameter. The larger the sum of the weight (between 0 and 1 by definition) the more important the parameter is, relative to the other parameters [4]. Input parameters were then ranked in terms of their importance to the quantities of interest.

The sensitivity analysis considered main effects and pairwise (first-order) interactions of input parameters on our quantities of interest.

### **Multi-model inference analysis**

We used multi-model inference with *glm* function from the *glmulti* R package [5], to estimate the relative importance of our input variables on our QOIs. Our first analysis considers the main effects of the input variables on the QOIs. Utilizing the default method of the *glmulti* package (exhaustive screening of all candidate models), we built all possible unique models based on the 11 input variables. Thus, the candidate set of models for each QOIs main effect analysis contained a total of 2048 models. We defined the confidence set of models as those models within two Akaike information criterion (AIC) unit difference (ΔAIC) from the best model [4] However, the *glmulti* function by default returns a confidence set of 100 models and delineates the number of models within 2 ΔAIC from the best model.

Our second analysis considers the pairwise interactions between our input parameters. We utilized the genetic algorithm approach of the *glmulti* package, to fit a sub-set of all possible unique models (due to the extremely large number of possible candidate models). The genetic algorithm approach randomly explores a subset of all possible models, with a bias towards better models to fit the best fitting model. Our confidence set is as defined above, i.e. within 2 ΔAIC of the best model. However, due to a large number of possible candidate models, the size of the returned confidence set was increased to 500 models (from the default of 100).

Note, before the multi-model inference fitting, we normalized the input parameters (by centering on zero and scaled to unit variance) to allow comparison of resulting model-averaged estimates on a common scale.

S1- S3 Tables (main effects) and S4 – S6 Tables (pairwise interactions), presents the top five models in the confidence sets for each our QOIs, their log likelihood values, number of fitted parameters (K), Akaike information criterion (AIC), AIC weights, and the goodness of fit (R2).

S7 Fig to S9 Fig (main effects) and S10 Fig to S12 Fig (pairwise interactions), shows the AIC profile of all fitted models.

S7 Table, presents the model averaged coefficients and relative-importance weights for the parameters from the best model with the pairwise interactions. The definition and description of the parameter symbols are as presented in Table 2 of the main article.

**S1 Table. Top five models in the confidence set exploring the main effects of input variables on** $\boldsymbol{MaxI}_{\boldsymbol{h}}$. The confidence set had 5 models within 2 ΔAIC from the best model.

| Model Order | Model | K | Log  Likelihood | AIC | AIC  weight | R2 |
| --- | --- | --- | --- | --- | --- | --- |
| 1 | ~ 1 +$t_{crit}$ + $T_{mean}$ + $T_{range}$+ $\beta_{h\to v}$ + $\beta_{v\to h}$ + $\mu_{v}$ + $R_{tot}$ | 7 | -990.96 | 1999.92 | 0.33 | 0.36 |
| 2 | ~ 1 +$t_{crit}$ + $T_{mean}$ + $T_{range}$ + $\beta_{h\to v}$ + $\beta_{v\to h}$ + $\mu_{v}$ + $\gamma_{v}$ + $R_{tot}$ | 8 | -990.45 | 2000.91 | 0.20 | 0.36 |
| 3 | ~ 1 +$t_{crit}$ + $T_{mean}$ + $T_{range}$ + $\beta_{v\to h}$ + $\mu_{v}$ + $iv$+ $R_{tot}$ | 7 | -990.91 | 2001.83 | 0.13 | 0.36 |
| 4 | ~ 1 +$t_{crit}$ + $T_{mean}$ + $T_{range}$ + $\beta_{v\to h}$ + $\beta_{v\to h}$+ $\mu_{v}$+ $q$ + $R_{tot}$ | 8 | -990.96 | 2001.92 | 0.12 | 0.36 |
| 5 | ~ 1 +$t_{crit}$ + $T_{mean}$ + $T_{range}$ + $\beta_{h\to v}$ + $\beta_{v\to h}$ + $\mu_{v}$ + $q$ + $R_{tot}$ | 8 | -990.40 | 2001.92 | 0.12 | 0.36 |


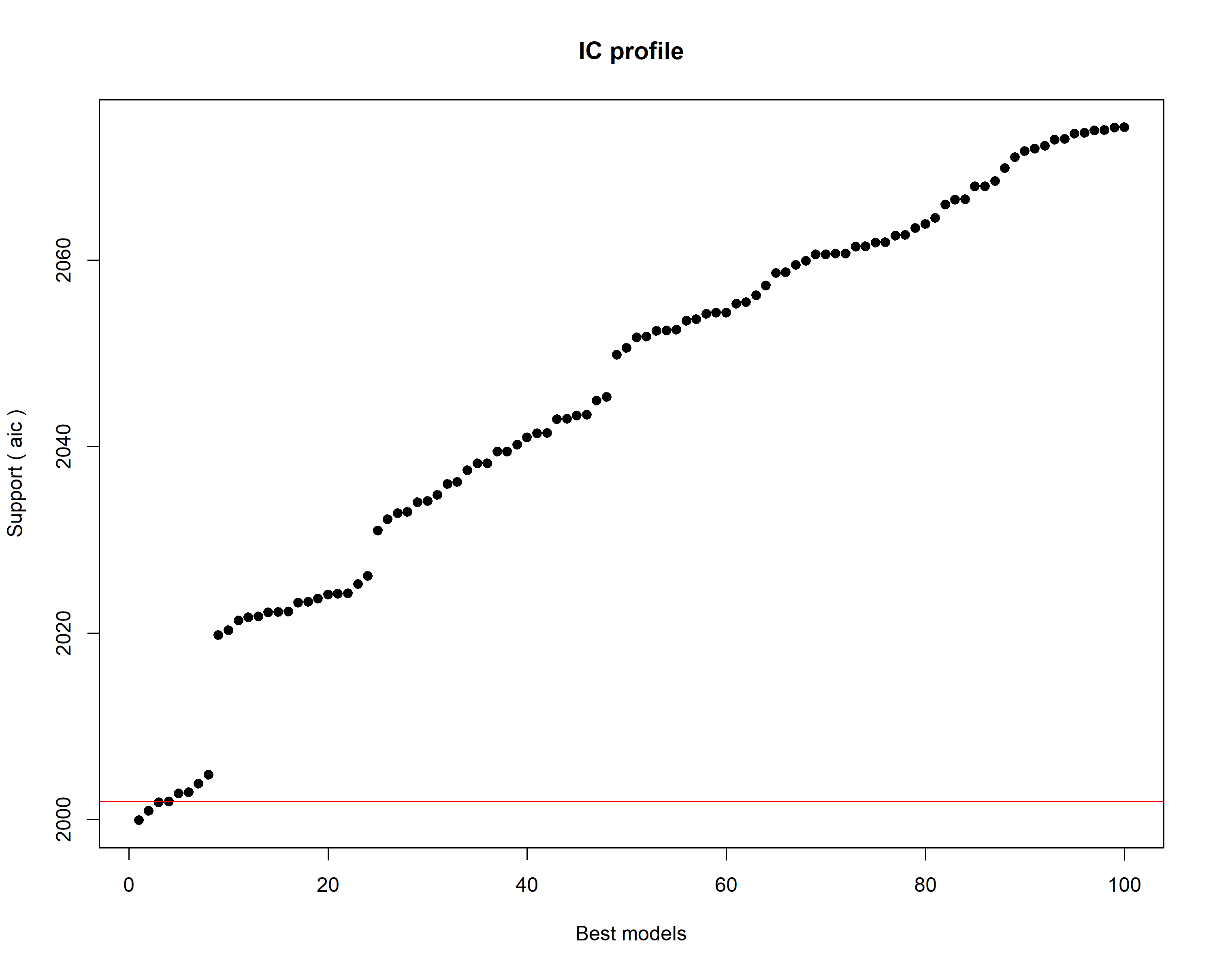


**S7 Fig. The AIC profile of models exploring the main effects of input variables on** $\boldsymbol{MaxI}_{\boldsymbol{h}}$. AIC values from the best to the worst model from the 100 returned models, horizontal line delineates models that are within 2 ΔAIC from the best model. Best AIC: 1999.92; Worst AIC: 2074.25

**S2 Table**. **Top five models in the confidence set exploring the main effects of input variables on** ${t\boldsymbol{maxI}}_{\boldsymbol{h}}$. The confidence set had 26 models within 2 ΔAIC from the best model.

| Model  Order | Model | K | Log  Likelihood | | AIC | AIC weight | R2 |
| --- | --- | --- | --- | --- | --- | --- | --- |
| 1 | ~ 1 +$t_{crit}$ + $T_{mean}$ + $\beta_{h\to v}$ + $\mu_{v}$ | 4 | 1295.92 | 2603.85 | | 0.04 | 0.31 |
| 2 | ~ 1 +$t_{crit}$ + $T_{mean}$ + $\beta_{h\to v}$ + $\mu_{v}$+ $R_{tot}$ | 5 | 1294.95 | 2603.91 | | 0.04 | 0.31 |
| 3 | ~ 1 +$t_{crit}$ + $T_{mean}$ + $\beta_{h\to v}$ + $\beta_{v\to h}$ + $\mu_{v}$ | 5 | 1295.08 | 2604.15 | | 0.03 | 0.31 |
| 4 | ~ 1 +$t_{crit}$ + $T_{mean}$ + $T_{range}$ + $\beta_{h\to v}$ + $\mu_{v}$ | 5 | 1295.14 | 2604.27 | | 0.03 | 0.31 |
| 5 | ~ 1 +$t_{crit}$ + $T_{mean}$ + $\beta_{h\to v}$ + $\beta_{v\to h}$ + $\mu_{v}$+ $R_{tot}$ | 6 | 1294.14 | 2604.29 | | 0.03 | 0.31 |

**
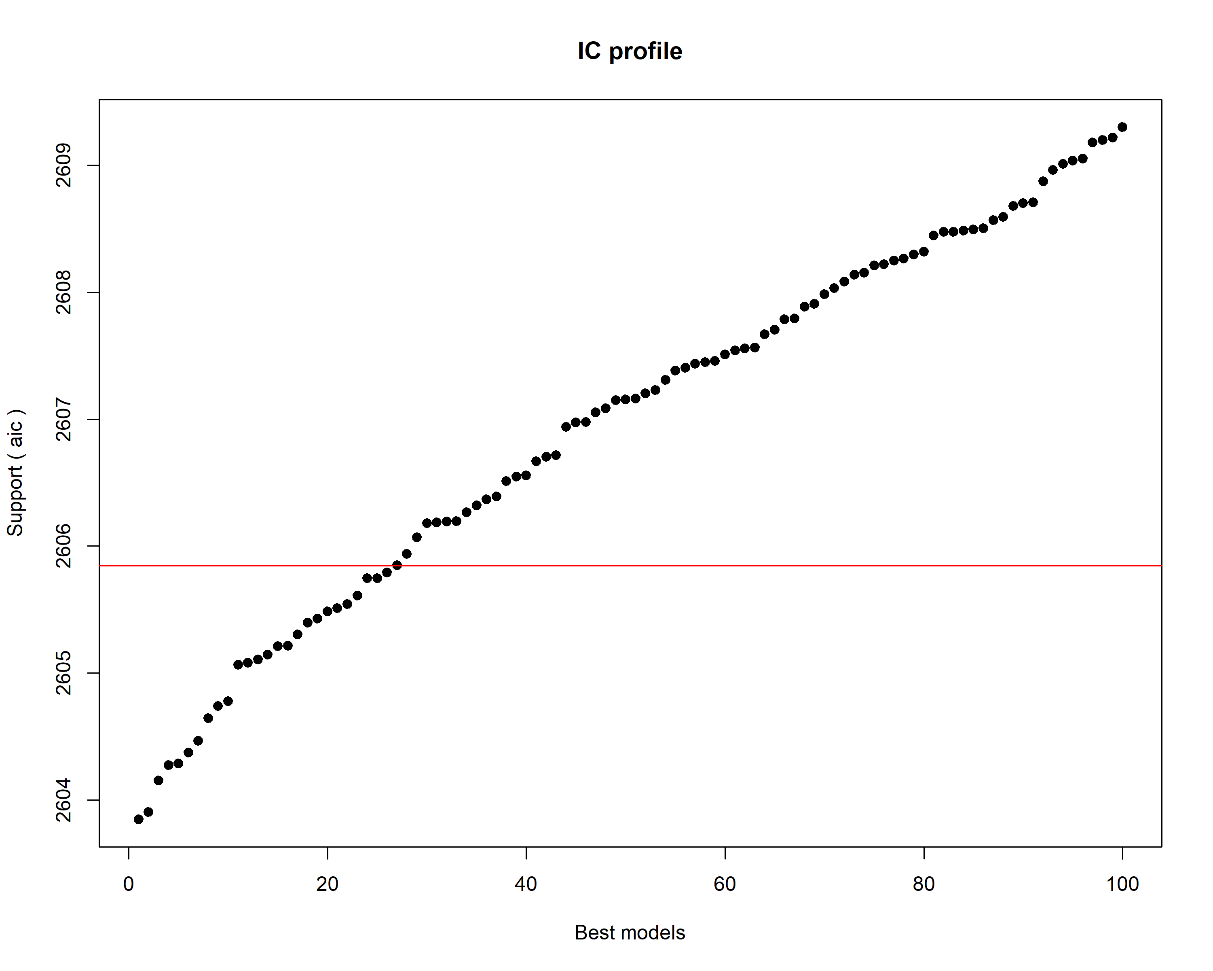
**

**S8 Fig. The AIC profile of models exploring the main effects of input variables on** $\boldsymbol{tmaxI}_{\boldsymbol{h}}$**.** AIC values from the best to the worst model from the 100 returned models, horizontal line delineates models that are within 2 ΔAIC from the best model. Best AIC: 2603.85; Worst AIC: 2609.30

**S3 Table**. **Top five models in the confidence set exploring main effects of input variables on** $\boldsymbol{cumI}_{\boldsymbol{h}}$. The confidence set had 5 models within 2 ΔAIC from the best model.

| Model  Order | Model | K | Log  Likelihood | AIC | AIC weight | R2 |
| --- | --- | --- | --- | --- | --- | --- |
| 1 | ~ 1 +$t_{crit}$ + $T_{mean}$ + $T_{range}$ + $\beta_{h\to v}$ + $\beta_{v\to h}$ + $\mu_{v}$ + $R_{tot}$ | 7 | -1207.91 | 2433.81 | 0.36 | 0.46 |
| 2 | ~ 1 +$t_{crit}$ + $T_{mean}$ + $T_{range}$ + $\beta_{h\to v}$ + $\beta_{v\to h}$ + $\mu_{v}$ + $\gamma_{v}$+ $R_{tot}$ | 8 | -1207.63 | 2435.25 | 0.17 | 0.46 |
| 3 | ~ 1 +$t_{crit}$+ $T_{mean}$ + $T_{range}$ + $\beta_{h\to v}$ + $\beta_{v\to h}$ + $\mu_{v}$ + $iv$+ $R_{tot}$ | 8 | -1207.90 | 2435.81 | 0.13 | 0.46 |
| 4 | ~ 1 +$t_{crit}+ T_{mean} + T_{range}$ + $\beta_{h\to v}$ + $\beta_{v\to h}$ + $\mu_{v}$ + $q$+ $R_{tot}$ | 8 | -1207.91 | 2435.81 | 0.13 | 0.46 |
| 5 | ~ 1 +$t_{crit}+ T_{mean} + T_{range}$ + $\beta_{h\to v}$ + $\beta_{v\to h}$ + $\mu_{v}$ + $R_{tot}$ | 7 | -1207.62 | 2435.81 | 0.13 | 0.46 |


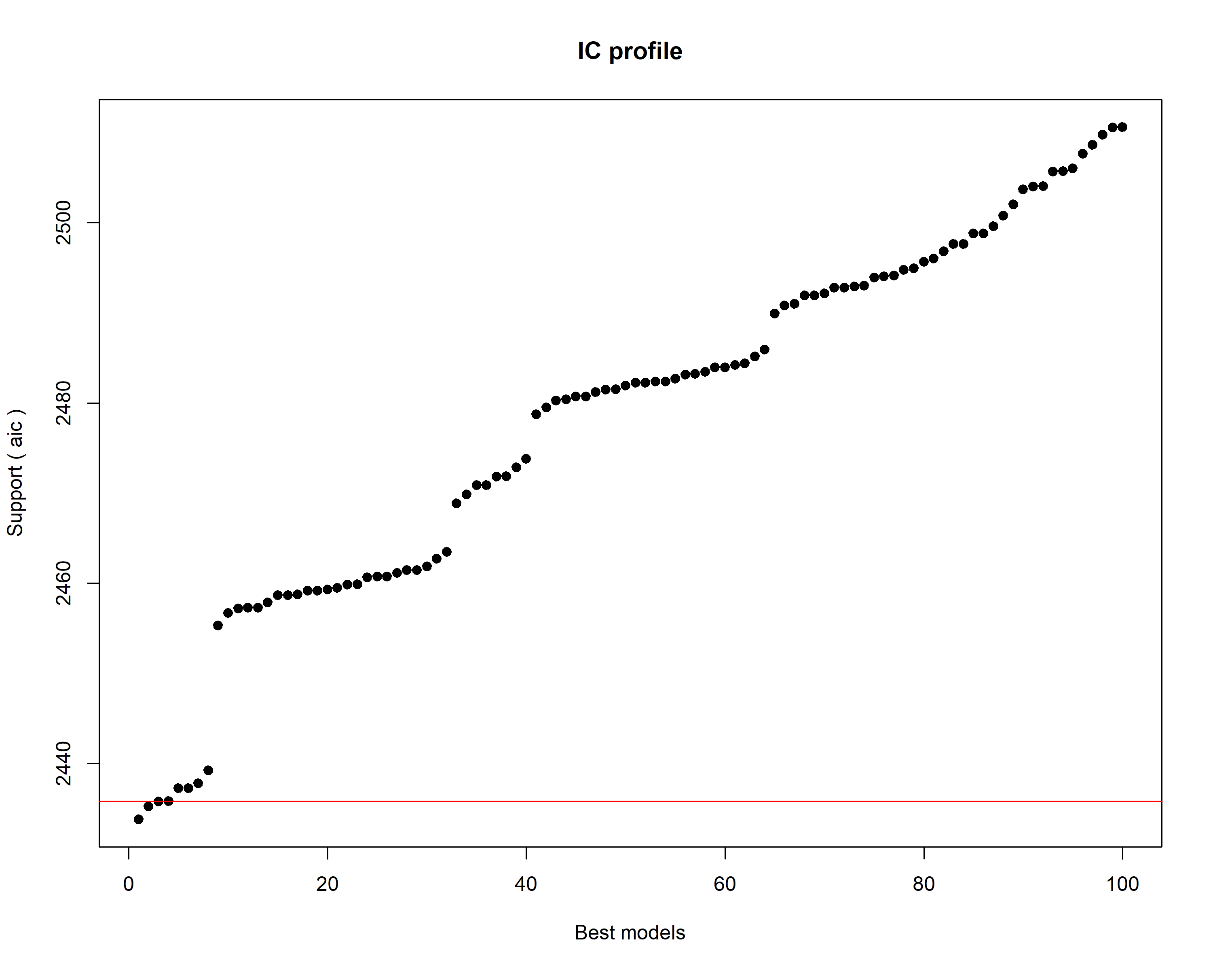


**S9 Fig. The AIC profile of models exploring the main effects of input variables on** $\boldsymbol{cumI}_{\boldsymbol{h}}$**.** AIC values from the best to the worst model from the 100 returned models, horizontal line delineates models that are within 2 ΔAIC from the best model. Best AIC: 2433.81; Worst AIC: 2510.59

**S4 Table**. **Top five models exploring in the confidence set the main effects and pairwise (first order) interactions of input variables on** $\boldsymbol{MaxI}_{\boldsymbol{h}}$. The confidence set had 47 models within 2 ΔAIC from the best model.

| Model Order | Model | K | Log Likelihood | AIC | AIC  weight | R2 |
| --- | --- | --- | --- | --- | --- | --- |
| 1 | ~ 1 + $\beta_{h\to v}$ + $\gamma_{v}$ + $iv$ +$t_{crit}:T_{mean}$ + $t_{crit}:T_{range}$ + $T_{mean}:T_{range}$ + $t_{crit}:\beta_{v\to h}$+ $\beta_{h\to v}: \mu_{v}$ + $T_{range}:\gamma_{v}$ + $\gamma_{v}:iv$ + $T_{range}:q$ + $T_{mean}:R_{tot}$+ $\beta_{v\to h}:R_{tot}$+ $q: R_{tot}$ | 14 | -958.11 | 1948.23 | 0.03 | 0.41 |
| 2 | ~ 1 + $\beta_{h\to v}$ + $\gamma_{v}$ + $iv$ + $t_{crit}:T_{mean}$+ $t_{crit}:T_{range}$+ $T_{mean}:T_{range}$ + $t_{crit}:\beta_{v\to h}$+ $\beta_{h\to v}: \mu_{v}$ + $T_{range}:\gamma_{v}$ + $\gamma_{v}:iv$ + $T_{range}:q$ + $T_{mean}:R_{tot}$+ $\beta_{v\to h}:R_{tot}$+ $\mu_{v}:R_{tot}$+ $q: R_{tot}$ | 15 | -957.45 | 1948.90 | 0.02 | 0.41 |
| 3 | ~ 1 + $\beta_{h\to v}$ + $\gamma_{v}$ + $iv$ + $t_{crit}:T_{mean}$ + $t_{crit}:T_{range}$+ $T_{mean}:T_{range}$ + $t_{crit}:\beta_{v\to h}$+ $t_{crit}:\mu_{v}$ + $\beta_{h\to v}: \mu_{v}$ + $T_{range}:\gamma_{v}$ + $\gamma_{v}:iv$ + $T_{range}:q$ + $T_{mean}:R_{tot}$+ $\beta_{v\to h}:R_{tot}$+ $q: R_{tot}$ | 15 | -957.55 | 1949.11 | 0.02 | 0.41 |
| 4 | ~ 1 + $\beta_{h\to v}$ + $\gamma_{v}$ + $iv$ + $t_{crit}:T_{mean}$ + $t_{crit}:T_{range}$ + $T_{mean}:T_{range}$+ $t_{crit}:\beta_{v\to h}$+ $T_{range}:\beta_{h\to v}$ + $\beta_{h\to v}: \mu_{v}$ + $T_{range}:\gamma_{v}$ + $\gamma_{v}:iv$ + $T_{range}:q$ + $T_{mean}:R_{tot}$+ $\beta_{v\to h}:R_{tot}$+ $q: R_{tot}$ | 15 | -957.57 | 1949.13 | 0.02 | 0.41 |
| 5 | ~ 1 + $\beta_{h\to v}$ + $\gamma_{v}$ + $iv$ + $t_{crit}:T_{mean}$ + $t_{crit}:T_{range}$ + $T_{mean}:T_{range}$ + $t_{crit}:\beta_{v\to h}$+ $t_{crit}:\beta_{v\to h}$ + $\beta_{h\to v}: \mu_{v}$ + $T_{range}:\gamma_{v}$ + $\gamma_{v}:iv$ + $T_{range}:q$ + $T_{mean}:R_{tot}$+ $T_{range}:R_{tot}$+ $\beta_{v\to h}:R_{tot}$ + $q: R_{tot}$ | 16 | -957.68 | 1949.36 | 0.02 | 0.41 |

**
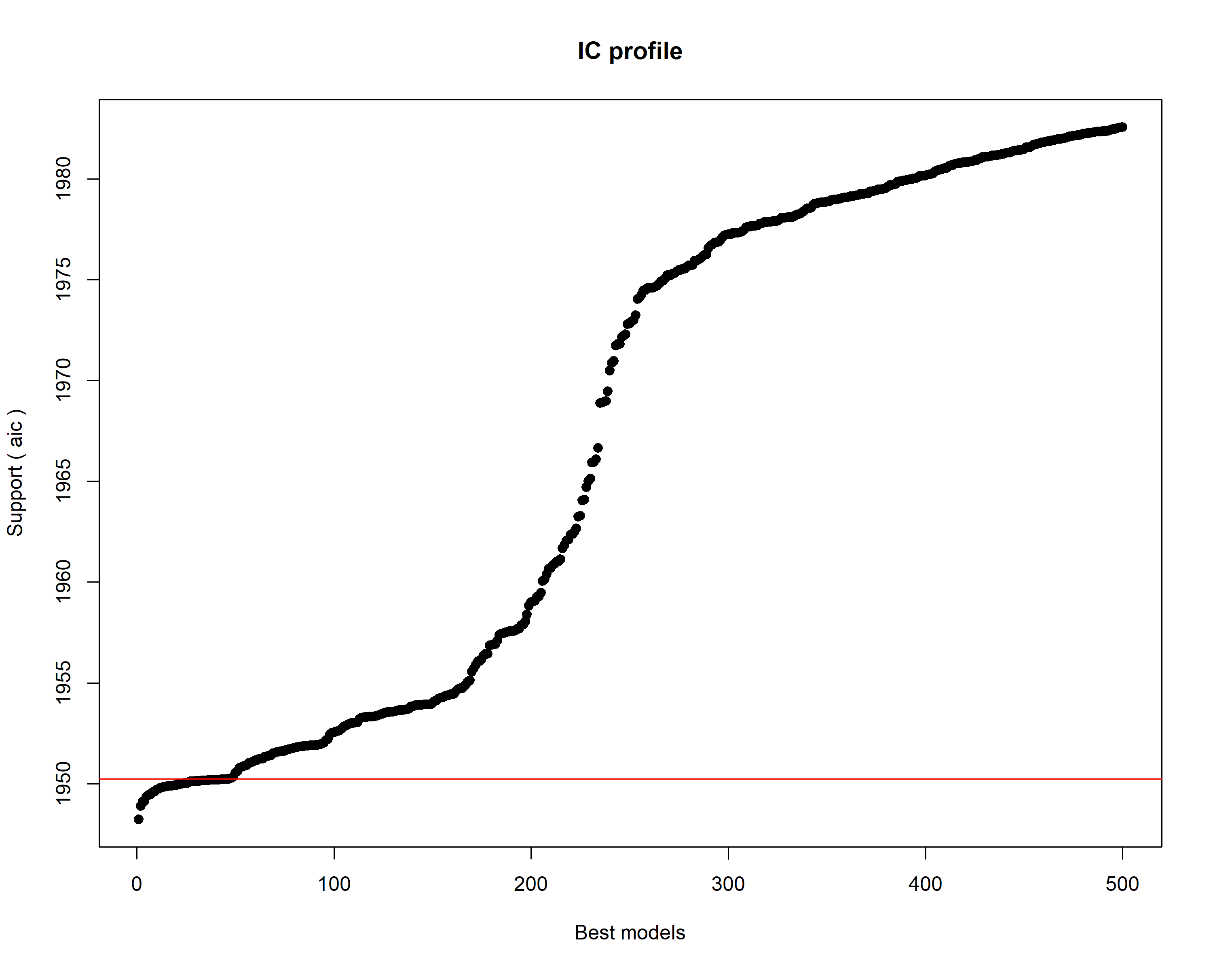
**

**S10 Fig. The AIC profile of models exploring the main effects of input variables on** $\boldsymbol{MaxI}_{\boldsymbol{h}}$**.** AIC values from the best to the worst model from the 500 returned models, horizontal line delineates models that are within 2 ΔAIC from the best model. Best AIC: 1948.23; Worst AIC: 1982.56

**S5 Table. Top five models in the confidence set exploring the main effects and pairwise (first order) interactions of input variables on** $\boldsymbol{tmaxI}_{\boldsymbol{h}}$**.** The confidence set had 128 models within 2 ΔAIC from the best model.

| Model Order | Model | K | Log  Likelihood | AIC | AIC  weight | R2 |
| --- | --- | --- | --- | --- | --- | --- |
| 1 | ~ 1 +$t_{crit}$ + $T_{mean}$ + $T_{range}$ + $\beta_{h\to v}$ + $\beta_{v\to h}$ + $\mu_{v}$ + $R_{tot}$+ $t_{crit}:T_{mean}$ + $t_{crit}:T_{range}$ + $T_{mean}:T_{range}$ + $T_{mean}:\beta_{h\to v}$ + $T_{range}:\beta_{h\to v}$ + $T_{mean}:\beta_{v\to h}$ + $\beta_{h\to v}:\beta_{v\to h}$ + $\beta_{v\to h}:\mu_{v}$ + $t_{crit}:\gamma_{v}$ + $\beta_{h\to v}:\gamma_{v}$ + $\beta_{v\to h}:\gamma_{v}$ + $\mu_{v}:\gamma_{v}$ + $t_{crit}:iv$ + $T_{range}:iv$ + $\beta_{v\to h}:iv$ + $t_{crit}:q$ + $T_{mean}:q$ + $iv:q$ + $T_{mean}:R_{tot}$+ $T_{range}:R_{tot}$+ $\beta_{h\to v}:R_{tot}$ + $\mu_{v}:R_{tot}$ | 29 | -1243.69 | 2547.38 | 0.01 | 0.40 |
| 2 | ~ 1 +$t_{crit}$ + $T_{mean}$ + $T_{range}$ + $\beta_{h\to v}$ + $\beta_{v\to h}$ + $\mu_{v}$ + $R_{tot}$+ $t_{crit}:T_{mean}$ + $t_{crit}:T_{range}$ + $T_{mean}:T_{range}$ + $T_{mean}:\beta_{h\to v}$ + $T_{range}:\beta_{h\to v}$ + $T_{mean}:\beta_{v\to h}$ + $\beta_{h\to v}:\beta_{v\to h}$ + $\beta_{v\to h}:\mu_{v}$ + $t_{crit}:\gamma_{v}$ + $\beta_{h\to v}:\gamma_{v}$ + $\beta_{v\to h}:\gamma_{v}$ + $\mu_{v}:\gamma_{v}$ + $t_{crit}:iv$ + $T_{range}:iv$ + $\beta_{v\to h}:iv$ + $t_{crit}:q$ + $T_{mean}:q$ + $iv:q$ + $T_{range}:R_{tot}$+ $\beta_{h\to v}:R_{tot}$ + $\mu_{v}:R_{tot}$ | 28 | -1244.74 | 2547.47 | 0.01 | 0.40 |
| 3 | ~ 1 +$t_{crit}$ + $T_{mean}$ + $T_{range}$ + $\beta_{h\to v}$ + $\beta_{v\to h}$ + $\mu_{v}$ + $R_{tot}$+ $t_{crit}:T_{mean}$ + $t_{crit}:T_{range}$ + $T_{mean}:T_{range}$ + $T_{mean}:\beta_{h\to v}$ + $T_{range}:\beta_{h\to v}$ + $T_{mean}:\beta_{v\to h}$ + $\beta_{h\to v}:\beta_{v\to h}$ + $\beta_{v\to h}:\mu_{v}$ + $t_{crit}:\gamma_{v}$ + $\beta_{h\to v}:\gamma_{v}$ + $\beta_{v\to h}:\gamma_{v}$ + $\mu_{v}:\gamma_{v}$ + $t_{crit}:iv$ + $T_{range}:iv$ + $\beta_{v\to h}:iv$ + $t_{crit}:q$ + $T_{mean}:q$ + $iv:q$ + $T_{mean}:R_{tot}$+ $T_{range}:R_{tot}$+ $\beta_{h\to v}:R_{tot}$ + $\mu_{v}:R_{tot}$ | 29 | -1244.75 | 2547.48 | 0.01 | 0.40 |
| 4 | ~ 1 +$t_{crit}$ + $T_{mean}$ + $T_{range}$ + $\beta_{h\to v}$ + $\beta_{v\to h}$ + $\mu_{v}$ + $R_{tot}$+ $t_{crit}:T_{mean}$ + $t_{crit}:T_{range}$ + $T_{mean}:T_{range}$ + $T_{mean}:\beta_{h\to v}$ + $T_{range}:\beta_{h\to v}$ + $T_{mean}:\beta_{v\to h}$ + $\beta_{h\to v}:\beta_{v\to h}$ + $\beta_{v\to h}:\mu_{v}$ + $t_{crit}:\gamma_{v}$ + $\beta_{h\to v}:\gamma_{v}$ + $\beta_{v\to h}:\gamma_{v}$ + $\mu_{v}:\gamma_{v}$ + $t_{crit}:iv$ + $T_{range}:iv$ + $\beta_{v\to h}:iv$ + $t_{crit}:q$ + $T_{mean}:q$ + $iv:q$ + $T_{mean}:R_{tot}$+ $T_{range}:R_{tot}$+ $\beta_{h\to v}:R_{tot}$ + $\mu_{v}:R_{tot}$ | 29 | -1244.77 | 2547.53 | 0.01 | 0.40 |
| 5 | ~ 1 +$t_{crit}$ + $T_{mean}$ + $T_{range}$ + $\beta_{h\to v}$ + $\beta_{v\to h}$ + $\mu_{v}$ + $R_{tot}$+ $t_{crit}:T_{mean}$ + $t_{crit}:T_{range}$ + $T_{mean}:T_{range}$ + $T_{mean}:\beta_{h\to v}$ + $T_{range}:\beta_{h\to v}$ + $T_{mean}:\beta_{v\to h}$ + $\beta_{h\to v}:\beta_{v\to h}$ + $\beta_{v\to h}:\mu_{v}$ + $t_{crit}:\gamma_{v}$ + $\beta_{h\to v}:\gamma_{v}$ + $\beta_{v\to h}:\gamma_{v}$ + $\mu_{v}:\gamma_{v}$ + $t_{crit}:iv$ + $T_{range}:iv$ + $\beta_{v\to h}:iv$ + $t_{crit}:q$ + $T_{mean}:q$ + $iv:q$ + $T_{mean}:R_{tot}$+ $T_{range}:R_{tot}$+ $\beta_{h\to v}:R_{tot}$ + $\mu_{v}:R_{tot}$ | 29 | -1244.79 | 2547.58 | 0.01 | 0.40 |

**
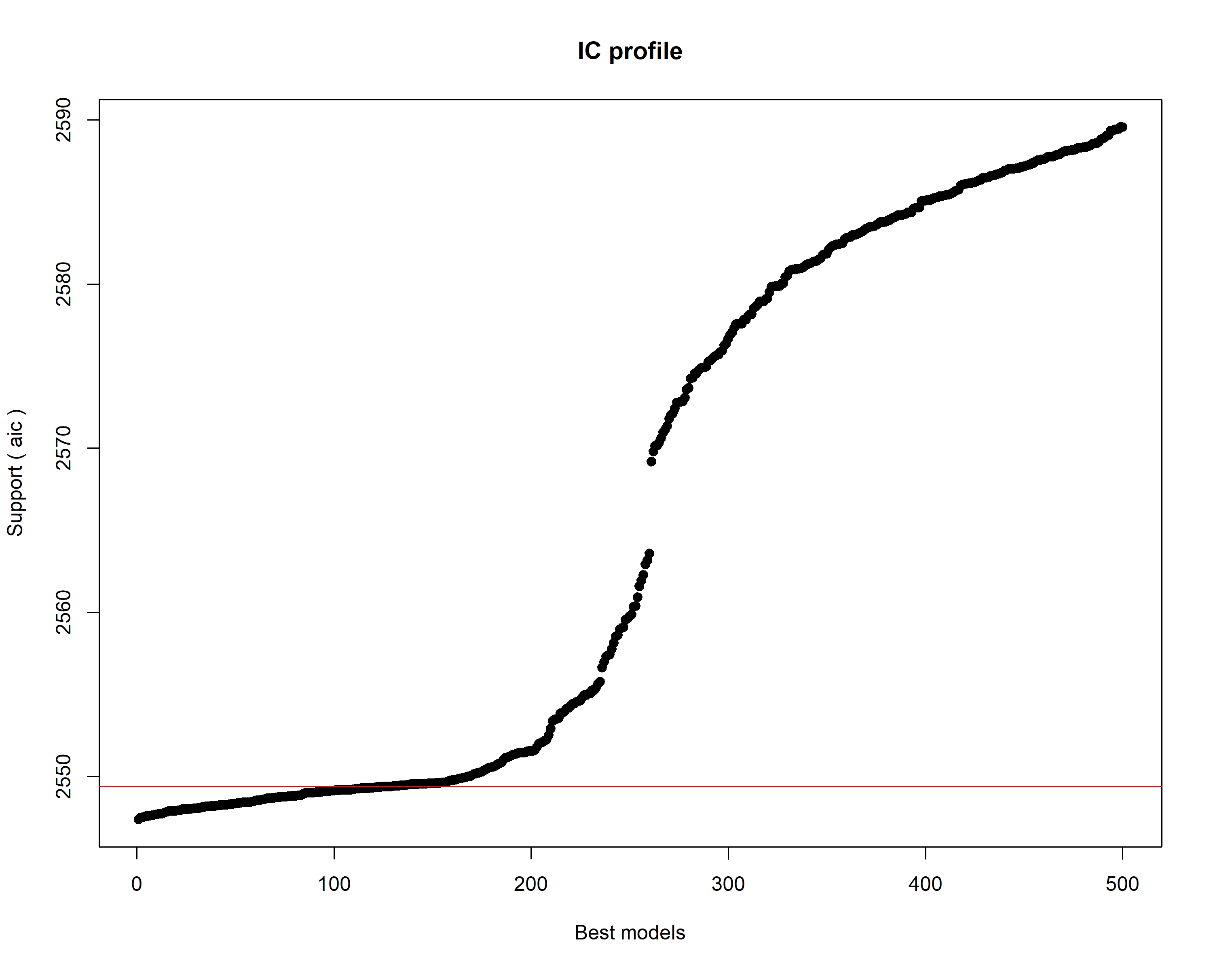
**

**S11 Fig. The AIC profile of models exploring main effects and pairwise (first order) interactions of input variables on** $\boldsymbol{tmaxI}_{\boldsymbol{h}}$**.** AIC values from the best to the worst model from the 500 returned models, horizontal line delineates models that are within 2 ΔAIC from the best model. Best AIC: 2547.38; Worst IC: 2589.56

**S6 Table**. **Top five models in the confidence set exploring the main effects and pairwise (first order) interactions of input variables on** $\boldsymbol{cumI}_{\boldsymbol{h}}$**.** The confidence set had 84 models within 2 ΔAIC from the best model.

| Model Order | Model | K | Log Likelihood | AIC | AIC weight | R2 |
| --- | --- | --- | --- | --- | --- | --- |
| 1 | ~ 1 +$t_{crit}$ + $T_{mean}$ + $T_{range}$ + $\beta_{h\to v}$ + $\beta_{v\to h}$ + $\mu_{v}$ + $R_{tot}$+ $t_{crit}:T_{mean}$ + $t_{crit}:T_{range}$ + $T_{mean}:T_{range}$ + $t_{crit}:\beta_{v\to h}$ + $t_{crit}:\mu_{v}$ + $\beta_{h\to v}: \mu_{v}$ + $t_{crit}:\gamma_{v}$ + $T_{range}:\gamma_{v}$ + $\gamma_{v}:iv$ + $t_{crit}:q$ + $iv:q$ + $T_{mean}:R_{tot}$ | 19 | -1159.61 | 2361.21 | 0.02 | 0.53 |
| 2 | ~ 1 +$t_{crit}$ + $T_{mean}$ + $T_{range}$ + $\beta_{h\to v}$ + $\beta_{v\to h}$ + $\mu_{v}$ + $R_{tot}$+ $t_{crit}:T_{mean}$ + $t_{crit}:T_{range}$ + $T_{mean}:T_{range}$ + $T_{range}:\beta_{v\to h}$ + $t_{crit}:\beta_{v\to h}$ + $t_{crit}:\mu_{v}$ + $\beta_{h\to v}: \mu_{v}$ + $t_{crit}:\gamma_{v}$ + $T_{range}:\gamma_{v}$ + $\gamma_{v}:iv$ + $t_{crit}:q$ + $iv:q$ + $T_{mean}:R_{tot}$ | 20 | -1158.63 | 2361.26 | 0.02 | 0.53 |
| 3 | ~ 1 +$t_{crit}$ + $T_{mean}$ + $T_{range}$ + $\beta_{h\to v}$ + $\beta_{v\to h}$ + $\mu_{v}$ + $R_{tot}$+ $t_{crit}:T_{mean}$ + $t_{crit}:T_{range}$ + $T_{mean}:T_{range}$ + $t_{crit}:\beta_{v\to h}$ + $t_{crit}:\mu_{v}$ + $\beta_{h\to v}: \mu_{v}$ + $t_{crit}:\gamma_{v}$ + $T_{range}:\gamma_{v}$ + $\beta_{h\to v}:iv$ + $\gamma_{v}:iv$ + $t_{crit}:q$ + $iv:q$ + $T_{mean}:R_{tot}$ | 20 | -1158.67 | 2361.34 | 0.02 | 0.53 |
| 4 | ~ 1 +$t_{crit}$ + $T_{mean}$ + $T_{range}$ + $\beta_{h\to v}$ + $\beta_{v\to h}$ + $\mu_{v}$ + $R_{tot}$+ $t_{crit}:T_{mean}$ + $t_{crit}:T_{range}$ + $T_{mean}:T_{range}$ + $T_{range}:\beta_{h\to v}$ + $t_{crit}:\beta_{v\to h}$ + $t_{crit}:\mu_{v}$ + $\beta_{h\to v}: \mu_{v}$ + $t_{crit}:\gamma_{v}$ + $T_{range}:\gamma_{v}$ + $\gamma_{v}:iv$ + $t_{crit}:q$ + $iv:q$ + $T_{mean}:R_{tot}$ | 20 | -1157.70 | 2361.41 | 0.01 | 0.53 |
| 5 | ~ 1 +$t_{crit}$ + $T_{mean}$ + $T_{range}$ + $\beta_{h\to v}$ + $\beta_{v\to h}$ + $\mu_{v}$ + $R_{tot}$+ $t_{crit}:T_{mean}$ + $t_{crit}:T_{range}$ + $T_{mean}:T_{range}$ + $T_{range}:\beta_{h\to v}$ + $t_{crit}:\beta_{v\to h}$ + $t_{crit}:\mu_{v}$ + $\beta_{h\to v}: \mu_{v}$ + $t_{crit}:\gamma_{v}$ + $T_{range}:\gamma_{v}$ + $\gamma_{v}:iv$ + $t_{crit}:q$ + $iv:q$ + $T_{mean}:R_{tot}$ | 20 | -1159.75 | 2361.51 | 0.01 | 0.53 |


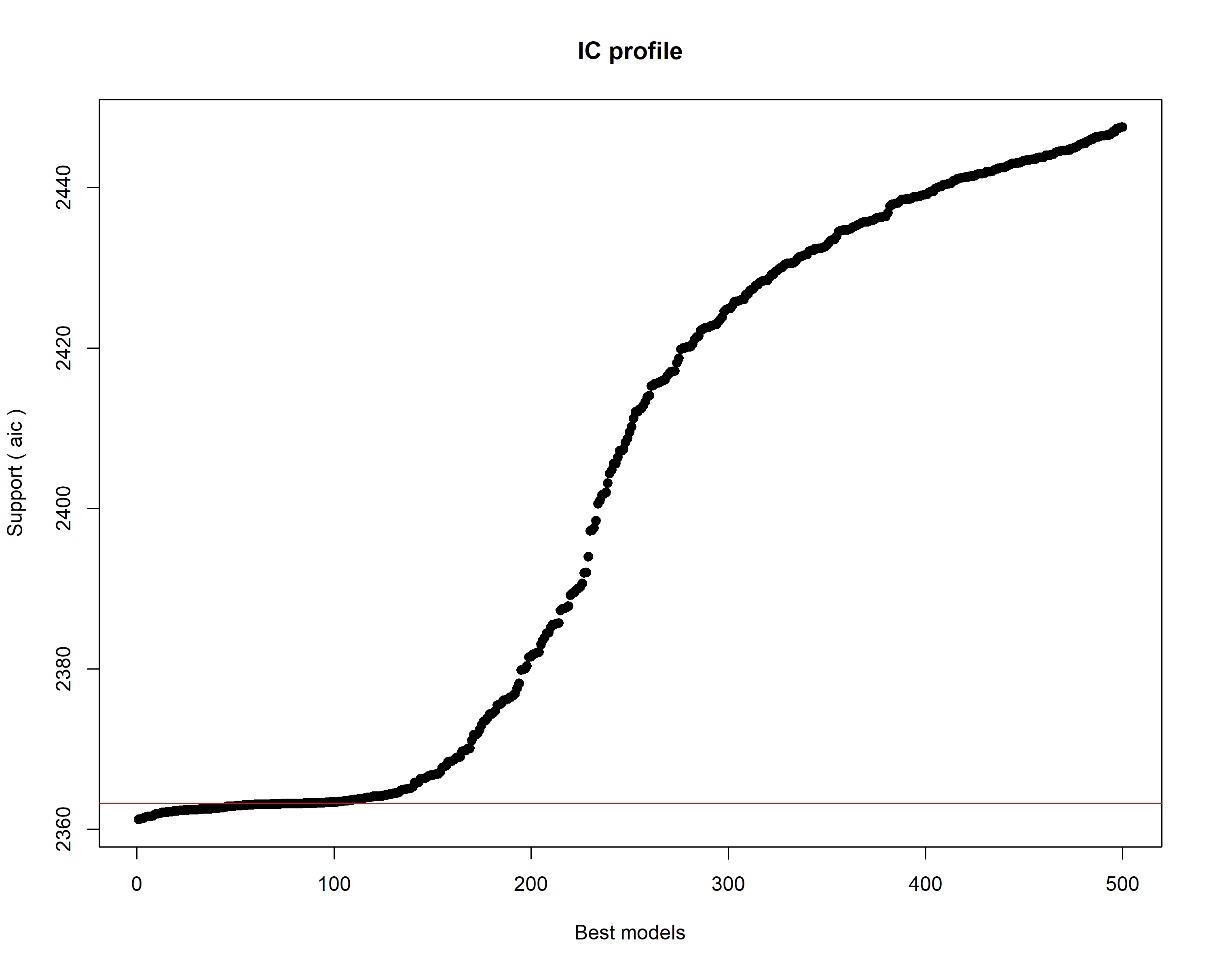


**S12 Fig. The AIC profile of models exploring main effects and pairwise (first order) interactions of input variables on** $\boldsymbol{cumI}_{\boldsymbol{h}}$. AIC values from the best to the worst model from the 500 returned models, horizontal line delineates models that are within 2 ΔAIC from the best model. Best AIC: 2361.21; Worst AIC: 2447.52

**S7 Table**. **Model averaged coefficients and relative-importance weights for dengue parameters** (from the best model with the pairwise interactions). Before model fitting, parameters were centered on zero and scaled to unit variance, to normalize parameters within the same range. Hence coefficient estimates are within the range of 0.0 to 1.0. Relative importance $\geq$0.90 appears in bold, an arbitrary cut-off for visualizing most important parameters.

| Parameter | $\boldsymbol{MaxI}_{\boldsymbol{h}}$ | |  | $\boldsymbol{tmaxI}_{\boldsymbol{h}}$ | |  | $\boldsymbol{cumI}_{\boldsymbol{h}}$ | |  |
| --- | --- | --- | --- | --- | --- | --- | --- | --- | --- |
|  | Coefficient^1^ | Relative  Importance | | Coefficient^1^ | Relative  Importance | | Coefficient^1^ | Relative  Importance | |
| $\boldsymbol{t}_{\boldsymbol{crit}}$ | 0.00 | 0.02 | | 0.43 | **1.00** | | 0.36 | **1.00** | |
| $\boldsymbol{t}_{\boldsymbol{crit}}\boldsymbol{:}\boldsymbol{T}_{\boldsymbol{mean}}$ | 0.38 | **1.00** | | 0.15 | **1.00** | | 0.10 | **1.00** | |
| $\boldsymbol{t}_{\boldsymbol{crit}}\boldsymbol{:}\boldsymbol{T}_{\boldsymbol{range}}$ | -0.14 | **0.96** | | 0.00 | 0.04 | | -0.07 | **1.00** | |
| $\boldsymbol{t}_{\boldsymbol{crit}}\boldsymbol{:}\boldsymbol{\beta}_{\boldsymbol{h\to v}}$ | 0.00 | 0.03 | | 0.00 | 0.01 | | 0.00 | 0.02 | |
| $\boldsymbol{t}_{\boldsymbol{crit}}\boldsymbol{:}\boldsymbol{\beta}_{\boldsymbol{v\to h}}$ | 0.16 | **0.98** | | 0.00 | 0.01 | | 0.07 | **1.00** | |
| $\boldsymbol{t}_{\boldsymbol{crit}}\boldsymbol{:}\boldsymbol{\mu}_{\boldsymbol{v}}$ | 0.00 | 0.02 | | 0.00 | 0.08 | | -0.07 | **1.00** | |
| $\boldsymbol{t}_{\boldsymbol{crit}}\boldsymbol{:}\boldsymbol{\gamma}_{\boldsymbol{v}}$ | 0.00 | 0.02 | | 0.05 | **0.99** | | 0.03 | 0.85 | |
| $\boldsymbol{t}_{\boldsymbol{crit}}\boldsymbol{:iv}$ | 0.00 | 0.01 | | -0.02 | 0.54 | | 0.00 | 0.03 | |
| $\boldsymbol{t}_{\boldsymbol{crit}}\boldsymbol{:q}$ | 0.00 | 0.02 | | 0.07 | **1.00** | | 0.04 | **0.97** | |
| $\boldsymbol{t}_{\boldsymbol{crit}}\boldsymbol{:}\boldsymbol{R}_{\boldsymbol{tot}}$ | 0.00 | 0.02 | | 0.00 | 0.02 | | 0.00 | 0.02 | |
| $\boldsymbol{T}_{\boldsymbol{mean}}$ | 0.00 | 0.02 | | 0.15 | **1.00** | | 0.15 | **1.00** | |
| $\boldsymbol{T}_{\boldsymbol{mean}}\boldsymbol{:}\boldsymbol{T}_{\boldsymbol{range}}$ | -1.04 | **1.00** | | 0.08 | **1.00** | | -0.10 | **1.00** | |
| $\boldsymbol{T}_{\boldsymbol{mean}}\boldsymbol{:}\boldsymbol{\beta}_{\boldsymbol{h\to v}}$ | 0.00 | 0.02 | | 0.07 | **1.00** | | 0.00 | 0.06 | |
| $\boldsymbol{T}_{\boldsymbol{mean}}\boldsymbol{:}\boldsymbol{\beta}_{\boldsymbol{v\to h}}$ | 0.00 | 0.02 | | -0.05 | **0.98** | | 0.00 | 0.02 | |
| $\boldsymbol{T}_{\boldsymbol{mean}}\boldsymbol{:}\boldsymbol{\mu}_{\boldsymbol{v}}$ | 0.00 | 0.02 | | 0.00 | 0.01 | | 0.00 | 0.02 | |
| $\boldsymbol{T}_{\boldsymbol{mean}}\boldsymbol{:}\boldsymbol{\gamma}_{\boldsymbol{v}}$ | 0.00 | 0.02 | | 0.00 | 0.01 | | 0.00 | 0.01 | |
| $\boldsymbol{T}_{\boldsymbol{mean}}\boldsymbol{:iv}$ | 0.00 | 0.02 | | 0.00 | 0.02 | | 0.00 | 0.02 | |
| $\boldsymbol{T}_{\boldsymbol{mean}}\boldsymbol{:q}$ | 0.00 | 0.02 | | 0.06 | **0.99** | | 0.00 | 0.02 | |
| $\boldsymbol{T}_{\boldsymbol{mean}}\boldsymbol{:}\boldsymbol{R}_{\boldsymbol{tot}}$ | 1.11 | **1.00** | | 0.04 | **0.94** | | 0.08 | **1.00** | |
| $\boldsymbol{T}_{\boldsymbol{range}}$ | 0.01 | 0.02 | | -0.04 | **0.96** | | -0.12 | **1.00** | |
| $\boldsymbol{T}_{\boldsymbol{range}}\boldsymbol{:}\boldsymbol{\beta}_{\boldsymbol{h\to v}}$ | -0.01 | 0.06 | | 0.07 | **0.99** | | -0.02 | 0.48 | |
| $\boldsymbol{T}_{\boldsymbol{range}}\boldsymbol{:}\boldsymbol{\beta}_{\boldsymbol{v\to h}}$ | 0.00 | 0.02 | | 0.00 | 0.03 | | 0.00 | 0.02 | |
| $\boldsymbol{T}_{\boldsymbol{range}}\boldsymbol{:}\boldsymbol{\mu}_{\boldsymbol{v}}$ | 0.00 | 0.02 | | 0.00 | 0.04 | | 0.00 | 0.02 | |
| $\boldsymbol{T}_{\boldsymbol{range}}\boldsymbol{:}\boldsymbol{\gamma}_{\boldsymbol{v}}$ | -0.49 | 0.99 | | 0.00 | 0.08 | | -0.04 | **0.96** | |
| $\boldsymbol{T}_{\boldsymbol{range}}\boldsymbol{:iv}$ | 0.00 | 0.01 | | 0.07 | **1.00** | | 0.00 | 0.03 | |
| $\boldsymbol{T}_{\boldsymbol{range}}\boldsymbol{:q}$ | 1.45 | **1.00** | | 0.00 | 0.01 | | 0.00 | 0.03 | |
| $\boldsymbol{T}_{\boldsymbol{range}}\boldsymbol{:}\boldsymbol{R}_{\boldsymbol{tot}}$ | 0.00 | 0.03 | | 0.04 | **0.95** | | 0.00 | 0.02 | |
| $\boldsymbol{\beta}_{\boldsymbol{h\to v}}$ | 1.15 | **0.99** | | 0.08 | **1.00** | | 0.23 | **1.00** | |
| $\boldsymbol{\beta}_{\boldsymbol{h\to v}}\boldsymbol{:}\boldsymbol{\beta}_{\boldsymbol{v\to h}}$ | 0.00 | 0.01 | | -0.05 | **0.99** | | 0.00 | 0.01 | |
| $\boldsymbol{\beta}_{\boldsymbol{h\to v}}\boldsymbol{:}\boldsymbol{\mu}_{\boldsymbol{v}}$ | -0.66 | **1.00** | | 0.00 | 0.04 | | -0.05 | **0.98** | |
| $\boldsymbol{\beta}_{\boldsymbol{h\to v}}\boldsymbol{:}\boldsymbol{\gamma}_{\boldsymbol{v}}$ | 0.00 | 0.01 | | -0.06 | **1.00** | | 0.00 | 0.01 | |
| $\boldsymbol{\beta}_{\boldsymbol{h\to v}}\boldsymbol{:iv}$ | 0.00 | 0.02 | | 0.00 | 0.02 | | 0.01 | 0.23 | |
| $\boldsymbol{\beta}_{\boldsymbol{h\to v}}\boldsymbol{:q}$ | 0.01 | 0.02 | | 0.00 | 0.02 | | 0.00 | 0.08 | |
| $\boldsymbol{\beta}_{\boldsymbol{h\to v}}\boldsymbol{:}\boldsymbol{R}_{\boldsymbol{tot}}$ | 0.00 | 0.02 | | -0.05 | **0.98** | | 0.00 | 0.03 | |
| $\boldsymbol{\beta}_{\boldsymbol{v\to h}}$ | 0.00 | 0.02 | | -0.06 | **0.99** | | 0.12 | **1.00** | |
| $\boldsymbol{\beta}_{\boldsymbol{v\to h}}\boldsymbol{:}\boldsymbol{\mu}_{\boldsymbol{v}}$ | 0.02 | 0.15 | | 0.05 | **0.98** | | 0.00 | 0.03 | |
| $\boldsymbol{\beta}_{\boldsymbol{v\to h}}\boldsymbol{:}\boldsymbol{\gamma}_{\boldsymbol{v}}$ | 0.00 | 0.02 | | -0.05 | **0.98** | | 0.00 | 0.05 | |
| $\boldsymbol{\beta}_{\boldsymbol{v\to h}}\boldsymbol{:iv}$ | 0.00 | 0.03 | | 0.01 | 0.36 | | 0.00 | 0.03 | |
| $\boldsymbol{\beta}_{\boldsymbol{v\to h}}\boldsymbol{:q}$ | 0.00 | 0.02 | | 0.00 | 0.01 | | 0.00 | 0.02 | |
| $\boldsymbol{\beta}_{\boldsymbol{v\to h}}\boldsymbol{:}\boldsymbol{R}_{\boldsymbol{tot}}$ | 0.16 | 0.85 | | 0.00 | 0.03 | | 0.00 | 0.02 | |
| $\boldsymbol{\mu}_{\boldsymbol{v}}$ | 0.00 | 0.02 | | -0.06 | **0.99** | | -0.25 | **1.00** | |
| $\boldsymbol{\mu}_{\boldsymbol{v}}\boldsymbol{:}\boldsymbol{\gamma}_{\boldsymbol{v}}$ | 0.00 | 0.03 | | 0.03 | 0.81 | | 0.00 | 0.03 | |
| $\boldsymbol{\mu}_{\boldsymbol{v}}\boldsymbol{:iv}$ | 0.00 | 0.02 | | 0.00 | 0.07 | | 0.00 | 0.03 | |
| $\boldsymbol{\mu}_{\boldsymbol{v}}\boldsymbol{:q}$ | 0.00 | 0.03 | | 0.00 | 0.02 | | 0.00 | 0.01 | |
| $\boldsymbol{\mu}_{\boldsymbol{v}}\boldsymbol{:}\boldsymbol{R}_{\boldsymbol{tot}}$ | -0.06 | 0.22 | | 0.05 | **0.97** | | 0.00 | 0.02 | |
| $\boldsymbol{\gamma}_{\boldsymbol{v}}$ | 1.27 | **1.00** | | 0.00 | 0.02 | | 0.00 | 0.04 | |
| $\boldsymbol{\gamma}_{\boldsymbol{v}}\boldsymbol{:iv}$ | -0.72 | **0.98** | | 0.00 | 0.02 | | -0.04 | **0.95** | |
| $\boldsymbol{\gamma}_{\boldsymbol{v}}\boldsymbol{:q}$ | 0.01 | 0.02 | | 0.00 | 0.02 | | 0.00 | 0.05 | |
| $\boldsymbol{\gamma}_{\boldsymbol{v}}\boldsymbol{:}\boldsymbol{R}_{\boldsymbol{tot}}$ | 0.01 | 0.03 | | 0.00 | 0.01 | | 0.00 | 0.05 | |
| $\boldsymbol{iv}$ | 0.53 | 0.65 | | 0.00 | 0.02 | | 0.00 | 0.01 | |
| $\boldsymbol{iv:}\boldsymbol{q}$ | 0.15 | 0.37 | | -0.05 | **0.99** | | -0.05 | **0.98** | |
| $\boldsymbol{iv:}\boldsymbol{R}_{\boldsymbol{tot}}$ | 0.01 | 0.03 | | 0.00 | 0.02 | | 0.00 | 0.03 | |
| $\boldsymbol{q}$ | -0.21 | 0.16 | | 0.01 | 0.21 | | 0.00 | 0.02 | |
| $\boldsymbol{q:}\boldsymbol{R}_{\boldsymbol{tot}}$ | -0.79 | **0.99** | | 0.00 | 0.01 | | 0.00 | 0.02 | |
| $\boldsymbol{R}_{\boldsymbol{tot}}$ | 0.02 | 0.02 | | 0.04 | **0.96** | | 0.13 | **1.00** | |

## **References**

1. Marino S, Hogue IB, Ray CJ, Kirschner DE. A methodology for performing global uncertainty and sensitivity analysis in systems biology. J Theor Biol. 2008;254(1):178-96. doi: <https://doi.org/10.1016/j.jtbi.2008.04.011>.

2. Wu J, Dhingra R, Gambhir M, Remais Justin V. Sensitivity analysis of infectious disease models: methods, advances, and their application. J R Soc Interface. 2013;10(86):20121018. doi: <https://doi.org/10.1098/rsif.2012.1018>.

3. Hamby DM. A review of techniques for parameter sensitivity analysis of environmental models. Environ Monit Assess. 1994;32(2):135-54. doi: <https://doi.org/10.1007/bf00547132>.

4. Burnham KP, Anderson DR. Model selection and multimodel inference: a practical information-theoretic approach: Springer, New York, NY; 2002.

5. Calcagno V, de Mazancourt C. Glmulti: an R package for easy automated model selection with (generalized) linear models. 2010. 2010;34(12): J Stat Softw. doi: <https://doi.org/10.18637/jss.v034.i12>.
